# Resveratrol Prevents Cellular and Behavioral Sensory Alterations in the Animal Model of Autism Induced by Valproic Acid

**DOI:** 10.3389/fnsyn.2018.00009

**Published:** 2018-05-22

**Authors:** Mellanie Fontes-Dutra, Júlio Santos-Terra, Iohanna Deckmann, Gustavo Brum Schwingel, Gustavo Della-Flora Nunes, Mauro Mozael Hirsch, Guilherme Bauer-Negrini, Rudimar S. Riesgo, Victorio Bambini-Júnior, Cecília Hedin-Pereira, Carmem Gottfried

**Affiliations:** ^1^Translational Research Group in Autism Spectrum Disorders (GETTEA), Universidade Federal do Rio Grande do Sul (UFRGS), Porto Alegre, Brazil; ^2^Department of Biochemistry, Universidade Federal do Rio Grande do Sul (UFRGS), Porto Alegre, Brazil; ^3^National Institute of Science and Technology on Neuroimmunomodulation (INCT-NIM), Oswaldo Cruz Institute, Oswaldo Cruz Foundation, Rio de Janeiro, Brazil; ^4^Department of Biochemistry, University of Buffalo, The State University of New York, New York, NY, United States; ^5^Child Neurology Unit, Clinical Hospital of Porto Alegre, Federal University of Rio Grande do Sul, Porto Alegre, Brazil; ^6^School of Pharmacology and Biomedical Sciences, University of Central Lancashire, Preston, United Kingdom; ^7^Institute of Biophysics Carlos Chagas Filho and Institute of Biomedical Sciences, Federal University of Rio de Janeiro, Rio de Janeiro, Brazil; ^8^VPPCB, Oswaldo Cruz Foundation (Fiocruz), Rio de Janeiro, Brazil

**Keywords:** animal model, GABA, inhibition, parvalbumin, resveratrol, sensory, synaptic proteins, VPA

## Abstract

Autism spectrum disorder (ASD) is characterized by impairments in both social communication and interaction and repetitive or stereotyped behaviors. Although its etiology remains unknown, genetic and environmental risk factors have been associated with this disorder, including the exposure to valproic acid (VPA) during pregnancy. Resveratrol (RSV) is an anti-inflammatory and antioxidant molecule known to prevent social impairments in the VPA animal model of autism. This study aimed to analyze the effects of prenatal exposure to VPA, as well as possible preventive effects of RSV, on sensory behavior, the localization of GABAergic parvalbumin (PV^+^) neurons in sensory brain regions and the expression of proteins of excitatory and inhibitory synapses. Pregnant rats were treated daily with RSV (3.6 mg/kg) from E6.5 to E18.5 and injected with VPA (600 mg/kg) in the E12.5. Male pups were analyzed in Nest Seeking (NS) behavior and in whisker nuisance task (WNT). At P30, the tissues were removed and analyzed by immunofluorescence and western blotting. Our data showed for the first time an altered localization of PV^+^-neurons in primary sensory cortex and amygdala. We also showed a reduced level of gephyrin in the primary somatosensory area (PSSA) of VPA animals. The treatment with RSV prevented all the aforementioned alterations triggered by VPA. Our data shed light on the relevance of sensory component in ASD and highlights the interplay between RSV and VPA animal model as an important tool to investigate the pathophysiology of ASD.

## Introduction

Autism spectrum disorder (ASD) is a highly prevalent neurodevelopmental condition affecting 1 in 68 children aged 8 years in the USA (American Psychiatry Association (APA), 2013) and is characterized, according to the DSM-5, by a behavioral dyad composed by impairments in communication and social interaction and repetitive or stereotyped behaviors (American Psychiatry Association (APA), 2013). Although many interesting theories have been recently proposed (Patterson, 2009; Lucchina and Depino, 2013; Sandin et al., 2014; Gottfried et al., 2015), the ASD etiology remains unknown. This in turn hinders the discovery of new biomarkers and treatments, making ASD a significant individual and societal challenge (Bambini-Junior et al., 2014a; Anderson, 2015; Hu et al., 2017; Masi et al., 2017).

Autism has a high genetic heritability, which can be demonstrated by the high agreement of ASD development in monozygotic twins (reaching values of up to 90%; Dietert et al., 2011), compared to a concordance rate of about 10% in dizygotic twins (Miles, 2011; Yoo, 2015). In addition, some environmental factors are also associated to ASD, including prenatal exposure to valproic acid (VPA; Rodier et al., 1997; Christensen et al., 2013; Smith and Brown, 2014). Thus, based on these clinical observations, an animal model of autism by prenatal exposure to VPA was developed. Since then, it has been extensively validated, demonstrating a myriad of behavioral (Schneider and Przewłocki, 2005; Haddad et al., 2009; Dendrinos et al., 2011; Favre et al., 2013; Gottfried et al., 2013; Roullet et al., 2013; Mabunga et al., 2015), molecular (Roullet et al., 2010; Gottfried et al., 2013), morphological (Rodier et al., 1997; Dendrinos et al., 2011; Favre et al., 2013; Gottfried et al., 2013) and electrophysiological autistic-like features (Dawson et al., 2005; Markram et al., 2008; Rinaldi et al., 2008).

Sensory impairments are one of the most prevalent comorbidities associated with ASD and are identified in more than 90% of patients (Geschwind, 2009). Indeed, hyper- or hyporeactivity to sensory input have been used as one of the four behavioral patterns observed to evaluate restricted, repetitive patterns of behavior, interests, or activities in ASD (American Psychiatry Association (APA), 2013). Common deficits include hyper-responsiveness to non-harmful stimuli (e. g., visual, tactile and auditory) and hypo-responsiveness to harmful (nociceptive) stimuli (American Psychiatry Association (APA), 2013). Studies investigating how sensory stimuli are processed and integrated in patients with ASD and animal models of autism are scarce. Yet, the impairments in the perception of the environment possibly affect both social and repetitive behaviors (Nienborg and Cumming, 2010; Dendrinos et al., 2011; Wöhr et al., 2015) and the sensory deficits can also be valuable for diagnostic purposes (Marco et al., 2011). The prompted Ayres proposed a Sensory integration (SI) theory to explain ASD and other neurological disorders (Cummins, 1991). Actually, previous studies showed correlations between sensory misprocessing in cortical and subcortical regions with altered excitatory/inhibitory balance and disorganization of cortical columnar and laminar pattern in autistic individuals (Spence and Schneider, 2009; Stoner et al., 2014; Khan et al., 2015).

Given the abnormal sensory behaviors present in the animal model of autism induced by VPA, we asked if the neuronal organization in the primary somatosensory area (PSSA) was affected by exposure to VPA. Interneurons have a major role in brain circuits and organization, acting as either switches or pattern generators and providing refinement to the countless connections present in the brain (Xu et al., 2010; Chu and Anderson, 2015). GABAergic neurons expressing parvalbumin (PV^+^-neurons) are the most common interneurons in the cortex, comprising 40% of the total interneuron population (Staiger et al., 2009; Xu et al., 2010; Rudy et al., 2011) and playing important roles in social memory, attention, integration of different sensory areas (Gogolla et al., 2009; Unichenko et al., 2017) and notably providing a relevancy filter in sensory processing (Yang et al., 2017). Therefore, we addressed the question of whether the PV^+^-neuron distribution was altered in somatosensory cortex and amygdala in the VPA model and if there was a concomitant change in inhibitory and excitatory synaptic markers in this region.

We recently showed that prenatal treatment with resveratrol (RSV), a polyphenol compound presenting antioxidant and anti-inflammatory properties, prevents altered social behavior in the VPA animal model of autism (Bambini-Junior et al., 2014b). Considering that the social impairments of the VPA animals could be explained, at least partially, by excitatory/inhibitory imbalance in the sensory cortices and amygdala, we asked if RSV could prevent these alterations.

## Materials and Methods

### Animals

Wistar rats were obtained from Center of Reproduction and Experimentation of Laboratory Animals (CREAL) and maintained under a standard 12/12-h light/dark cycle (light cycle starting at 7 am and ending at 7 pm) at a constant temperature of 22 ± 1°C. The animals had *ad libitum* access to food and water, and were handled in accordance with the guidelines established by the National Council for the Control of Animal Experimentation (CONCEA) of Brazil. This project was approved by the ethics committee of the Federal University of Rio Grande do Sul (CEUA-UFRGS #31872) and by the Clinical Hospital of Porto Alegre (HCPA-FIPE #160477).

Animals were mated overnight and pregnancy was verified by next morning through presence of spermatozoa in the vaginal smear. This was considered the embryonic day 0.5 (E0.5). Pregnant rats were divided into four groups according to the treatment they received: Control, RSV, VPA, or RSV+VPA. From E6.5 to E18.5, the pregnant females received a daily subcutaneously injection of RSV (Fluxome, Stenløse, Denmark) at 3.6 mg/kg or dimethyl sulfoxide (DMSO, equivalent volume of RSV injection) as previously described (Bambini-Junior et al., 2014b). On E12.5, rats received a single intraperitoneal injection with either VPA at 600 mg/kg (Acros Organics, NJ, USA) or saline solution 0.9%.

### Behavioral Tasks

#### Nest Seeking Behavior

We assessed the nest-seeking (NS) response mediated by olfactory discrimination as described previously (Schneider and Przewłocki, 2005) at the postnatal day 10 (P10). All litter (males and females) was evaluated, since sex is very difficult to determine during behavioral tests before P10 and one would have to manipulate the litter, which could introduce a stress component. The apparatus used was a plastic container (30 × 20 × 13 cm) that was divided in thirds and had the lateral sections covered with wood shavings, but leaving a clear uncovered center. One side is filled with the home-cage bedding (nest shavings) and in the other side with sterilized shavings. The pup was placed in the center of the apparatus and the latency to reach the nest shavings and the time to make any choice was registered. The total time of the test was limited to 60 s. In order to maintain the smell of the litter/mother, we did not change the shavings in the home-cage in the 2 days preceding the test.

#### Whisker Nuisance Task (WNT)

**Figure 1:**
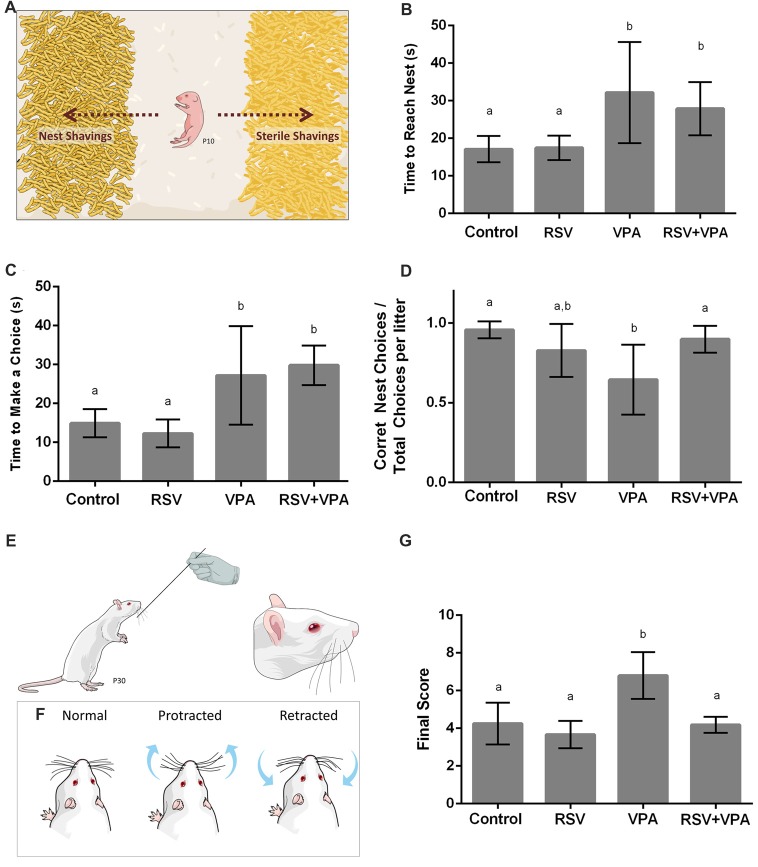
Animals from the valproic acid (VPA) model of autism demonstrate impaired sensorial behavior, which is counteracted by resveratrol (RSV). **(A)** Representative illustration from the Nest Seeking (NS) test. RSV administration could not prevent all behavioral alterations in VPA groups, which presented an increasing time to reach the nest **(B)** and increased latency to make any choice **(C)**. Whilst VPA induced significant decrease in the accuracy of the choice, prenatal RSV treatment prevented this feature **(D)**. **(E,F)** Representative illustration of the stimulation in the Whisker Nuisance Task (WNT; **E**) and possible position of whiskers: protracted (curiosity), retracted (avoidance) or not showing any particular response **(F)**. **(G)** Behavioral analysis of WNT, demonstrating the hyper-responsiveness of VPA animals, a behavior prevent by RSV treatment. Different letters indicate statistically significant differences between groups. Values plotted are Mean ± standard deviation (SD). Statistical analysis: ordinary one-way analysis of variance (ANOVA) followed by Bonferroni.

During this test, the animal behavioral response to direct vibrissae stimulation was observed in P30 animals. All tests and analyses were performed blindly. Since this is a sensory test, prior to testing, animals were familiarized with handling of the experimenter and habituated with the empty housing (57.1 × 39.4 × 15.2 cm) coated with an absorbent pad. To perform the test, the vibrissae are stimulated with a wooden toothpick for three consecutive periods of 5 min (15 min in total) with a 30 s interval between stimulation (Figures 1E,F). Animals were scored according to a scale developed by McNamara et al. (2010), in which freezing, stance and body position, breathing, whisker position, whisking response, evading stimulation, response to stick presentation and grooming are classified from 0 to 2 according to the response (0 = absent/typical, 1 = present/light response and 2 = profound/accentuated response; Supplementary Table S1). The sum of all scores is then calculated. Low scores (0–4) indicate normal responses, in which the animal is calm or indifferent to stimulation. High scores (8–16) indicate abnormal responses to stimulation, in which the animal freezes, shakes, or is aggressive (McNamara et al., 2010).

### Tissue Preparation and Analysis

#### Transcardiac Perfusion

P30 male rats were anesthetized (75 mg/kg ketamine +10 mg/kg xylazine) and subjected to transcardiac perfusion with 0.9%-NaCl solution followed by 1.5%-paraformaldehyde and 4%-paraformaldehyde solutions before the removal of the brain. The tissues were post-fixed for 4 h in a 4%-paraformaldehyde solution and subsequently cryoprotected by sequential immersion in 15% and 30%-sucrose solutions in PBS (the tissue was kept in each solution until complete submersion).

#### Immunofluorescence

Brains were embedded in Tissue-Tek^®^ and kept in −80°C ultra-freezer until further processing. Coronal brain slices (25 μm) were obtained in cryostat (Leica Microsystems GmbH) and a rat brain atlas (Paxinos and Watson, 1997) was utilized to identify sections containing the PSSA and the amygdala. We obtained the slices both from PSSA and amygdalar region according to the Paxinos rat atlas, using the following markers: Bregma (anteroposterior): −3.48 mm, interaural: 5.52 mm (Figure 62 from Paxinos rat atlas). After the immunofluorescence protocol from these slices described above, we delimited the regions as it follows: for PSSA we first localized the CA2 hippocampal region. Then moved laterally from this region until finding cortical border, where we can find the barrel fields, a specific region for whisker sensory processing. We localized the layer II/III and IV/V examining the cytoarchitecture of neuronal composition positive to NeuN labeling, as described in literature (Narayanan et al., 2017). For amygdalar region, we first localized the external capsule, a white matter region easily identified by DAPI staining. Then, we moved dorsoventrally to the end of this white matter region and, medially to this point, we identified lateral amygdalar region. We consider the amygdalar region since the histological contour of lateral amygdalar complex is difficult to determine.

The immunostaining procedure was performed in the following steps: (1) exposure to vapors of 4%-paraformaldehyde (10 min); (2) three washes with PBS 0.1 M buffer (5 min each); (3) permeabilization with PBS-Triton 0.1% (10 min); (4) three washes with PBS 0.1 M buffer (5 min each); (5) antigen retrieval suing citrate buffer at 60°C (1 h); (6) two washes with PBS-Triton 0.1% (5 min each); (7) blocking with BSA 5% in PBS-Triton 0.1% (1 h); (8) incubation with primary antibodies—diluted to 1:500 in blocking solution—for 48 h at 4°C; (9) five washes with PBS 0.1 M buffer (3 min each); (10) incubation with both secondary antibodies anti-mouse and anti-rabbit—diluted to 1:2000 in blocking solution) for 2 h at room temperature; (11) five washes with PBS 0.1 M buffer (3 min each); (12) incubation with DAPI solution (10 min); and (13) five washes with PBS 0.1 M buffer (3 min each) followed by addition of mounting medium with fluorshield and coverslip. The list of antibodies used in this work is available in the Supplementary Table S2.

Images were obtained with at least eight times per image (dimension: 635.9 × 635.9 microns) in a confocal microscope (Olympus FluoView FV1000 confocal laser scanning) of the Electron Microscopy Core. Processing and quantification of all tomes obtained from two to four tissue sections per glass slide were performed using the ImageJ software with the Cell Counter plug-in. The neuronal quantification results are shown in absolute number of NeuN^+^ labeled cells (total neuronal cells), PV^+^ and NeuN^+^ labeled cells (PV-neuronal cells) and in density of PV^+^-neuronal cells (which is the ratio between absolute number of PV^+^-neuronal cells by the total number of neuronal cells) for layer II/III, layer IV/V and all layers (II/III and IV/V). The number of PV^+^-neurons, total neurons and total cells were counted by an observer blind to the animal group.

### Eletrophoresis and Western Blotting

Amygdala region (AmR) and PSSA were surgically isolated and fresh tissues homogenates were prepared in lysis buffer. Protein concentration was measured by Lowry method (Lowry et al., 1951), equal amounts of protein (40 μg) were loaded in SDS-polyacrylamide gels and transferred to nitrocellulose membranes. After overnight incubation with the primary antibody at 4°C (Anti-PSD95, Anti-gephyrin, Anti-synaptophysin or Anti- β-actin; Supplementary Table S3), membranes were incubated with the proper secondary antibody conjugated to HRP (Donkey anti-mouse-IgG HRP or Goat anti-rabbit-IgG HRP) at room temperature for 1:30 h. No stripping of the membranes was performed (Supplementary Figures S1, S2). The SuperSignal West Pico reagent (Thermo Fisher Scientific) was used and its chemiluminescence was detected using the ImageQuant LAS 4000 immunodetector (GE HealthCare Life Sciences).

### Statistical Analysis

One-way analysis of variance (ANOVA) followed by Bonferroni’s *post hoc* multi comparison test was performed using the IBM SPSS software (version 20.0). Data are reported as mean ± standard deviation (SD), considering significant when *p* < 0.05. In graphic representation, different letters indicate statistically significant differences between the experimental groups. The total number of animals analyzed in each experiment was four animals from at least four different litters per group.

## Results

### In the Nest Seeking Behavior, RSV Prevents the Reduction of Accuracy But Not the Increase in Latency for Choice Induced by VPA

In order to evaluate the effects of VPA and RSV on olfactory discrimination, P10 pups were tested for NS behavior (Figure 1A). The following parameters were evaluated: latency to reach the nest shavings, latency to make any choice (nest or sterile shavings) and percentage of correct choices (reach the nest shavings) per litter. The latency to reach the nest shavings (Figure 1B) was increased in animals of the groups VPA (*p* = 0.0063) and RSV+VPA (*p* = 0.0486) when compared to the control group (Control: 17.1 ± 3.4, RSV: 17.4 ± 3.2, VPA: 32.1 ± 13.4, RSV+VPA: 27.8 ± 7.0, *F* = 6.8). In a similar way, the latency to make any choice (Figure 1C) was also increased in VPA (*p* = 0.0116) and RSV+VPA (*p* = 0.0011) groups compared to the control (Control: 14.8 ± 3.6, RSV: 12.2 ± 3.5, VPA: 27.2 ± 12.6, RSV+VPA: 29.8 ± 5.1, *F* = 13.05). Thus, VPA delayed the general response time of the animals, which was not prevented by RSV. There were no significant differences between groups in latency to reach sterile shavings and in the total time spent by the pups in either of the shavings (data not shown). However, prenatal administration of RSV successfully prevented the reduction of percentage of correct choices (Figure 1D) seen in the VPA group (Control: 0.95 ± 0.05, RSV: 0.8 ± 0.16, VPA: 0.6 ± 0.22, RSV+VPA: 0.89 ± 0.08, *F* = 5.9; *p* = 0.0020, VPA vs. Control and *p* = 0.0252, VPA vs. RSV+VPA).

### RSV Prevents the Abnormal Response to Direct Whisker Stimulation Observed in Rats of the VPA Model of Autism

Since ASD is usually associated with several impairments in SI, we sought to investigate the behavioral response of rats of the VPA model of autism in the Whisker Nuisance Task (WNT; Figure 1E). Animals from the VPA group increased WNT scores (Figure 1G) as compared to the control group (*p* = 0.0027, VPA compared to the control group). Strikingly, prenatal treatment with RSV averted the over-responsiveness induced by VPA (Control: 4.2 ± 1.1, RSV: 3.6 ± 0.72, VPA: 6.8 ± 1.2, RSV+VPA: 4.2 ± 0.42, *F* = 12.4; *p* = 0.0006, VPA vs. RSV+VPA group).

### RSV Counteracts the VPA Effects on Neuronal Organization in the Primary Somatosensory Cortex, Promoting Typical Laminar Distribution and Localization of PV^+^-Neurons

**Figure 2:**
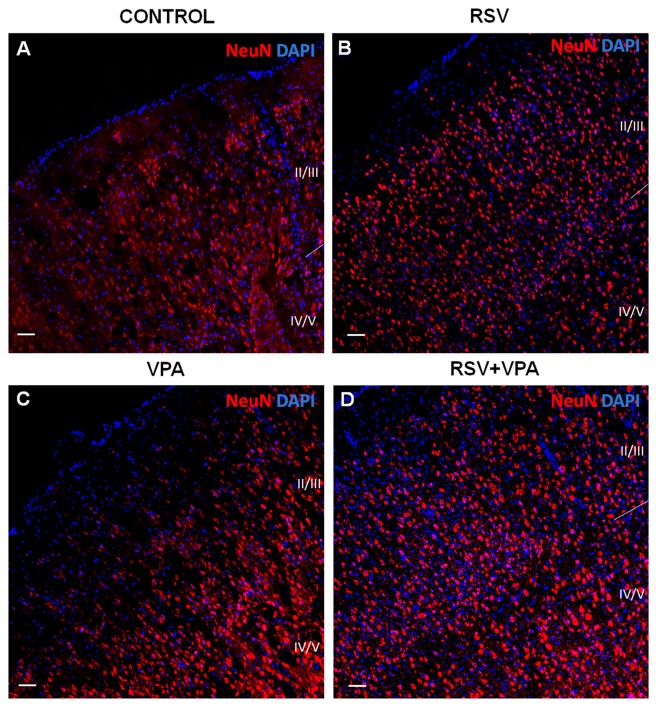
RSV prevents the impairments in cortical organization of the primary somatosensory area (PSSA) induced by VPA. **(A–D)** Cell nuclei—blue (DAPI) and total neurons—red (NeuN^+^). Layers II-III and IV-V can be distinctly visualized by its specific cellularity, enabling a qualitative analysis of the cortical laminar organization between the groups. Scale bar = 50 μm.

As shown in Figures 2A,B, the control and RSV groups presented a typical cortical organization, with a high number of medium-sized pyramidal neurons in layer II-III and granular and largest pyramidal neurons in layer IV-V, and a low cellularity between these layers. However, the VPA exposure induced visible alterations in cellular organization, increasing the space between layer I and layer II-III while drastically reducing the spacing between layer II-III and layer IV-V (Figure 2C). Interestingly, prenatal RSV treatment was able to prevent the VPA effects on cortical organization (Figure 2D).

**Figure 3:**
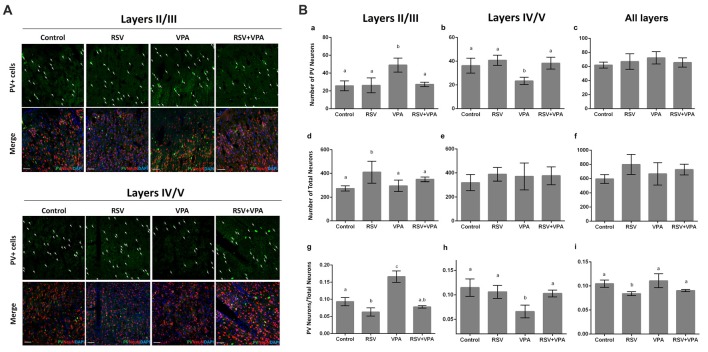
RSV averts the abnormal distribution of PV+ GABAergic neurons in the PSSA of animals of the VPA model of autism. Cell nuclei—blue (DAPI), total neurons—red (NeuN^+^) and GABAergic parvalbumin (PV^+^) neurons—green. **(A)** Representative immunofluorescence images of PSSA. Layer II-III and IV-V are separated in order to improve visualization of cell distribution. In each case, the first micrograph presents the green channel showing PV^+^ cells distribution in the indicated groups, followed by the merge image of three channels below (green, red and blue). **(Ba–i)** Quantitative analysis of PV^+^ cells in layer II-III and IV-V. Different letters indicate statistically significant differences between groups. Values plotted are Mean ± SD. Statistical analysis: ordinary one-way ANOVA followed by Bonferroni. Scale bar = 50 μm.

We then asked if there was any change in the number of PV^+^- neurons in layer II-III and layer IV-V of the PSSA. Representative micrographs of the layer II-III (a–d) and layer IV-V (e–h) are shown in Figure 3A. Our quantitative analysis revealed a significant increase in number of PV^+^ neurons in layer II-III of the VPA group, when compared to control animals (*p* = 0.0029). In addition, as showed in Figure 3Ba, RSV treatment successfully prevented this alteration (Control: 25.7 ± 5.5, RSV: 26.2 ± 8.3, VPA: 49 ± 7.8, RSV+VPA: 27.3 ± 2.5, *F* = 11.1; *p* = 0.0087, VPA compared to RSV+VPA group). On the other hand, animals of the VPA group showed reduced numbers (*p* = 0.0180) of PV^+^-neurons in layer IV-V, as compared to the control group (Figure 3Bb). Interestingly, RSV was also able to prevent this change (Control: 36.2 ± 6.3, RSV: 40.7 ± 4.2, VPA: 23.3 ± 3.3, RSV+VPA: 38.3 ± 5.03, *F* = 10.2; *p* = 0.0110, VPA compared to the RSV+VPA group). No significant differences were found in the sum of PV^+^-neurons of all cortical layers, suggesting an impairment of neuron localization rather than overall quantity (Figure 3Bc).

We also evaluated the total number of neurons in layer II-III and layer IV-V of PSSA using immunostaining with the NeuN marker. Our results pointed out for an effect of RSV exposure (*p* = 0.0328), increasing the total number of neurons in layer II-III (Figures 3Bd–f), in comparison to the control group (Control: 274.3 ± 21.8, RSV: 410.5 ± 91.9, VPA: 296.3 ± 47.5, RSV+VPA: 350 ± 19.9, *F* = 4.7).

When normalizing the number of PV^+^-neurons to the total number of neurons, the VPA group presented a significantly increased ratio in layer II/III (*p* < 0.0001), which was prevented by RSV (*p* < 0.0001, VPA compared to the RSV+VPA group). Surprisingly, the RSV treatment *per se* reduced the ratio PV^+^-neurons/total neurons (Figure 3Bg; *p* = 0.0356) in this same region when compared to control (Control: 0.09319 ± 0.01211, RSV: 0.063 ± 0.01, VPA: 0.16 ± 0.016, RSV+VPA: 0.07 ± 0.003, *F* = 52.4). We also observed a significant decrease in the PV^+^-neurons/total neurons ratio in the VPA group compared to controls (Figure 3Bh; *p* = 0.0023). Importantly, this alteration was also prevented by RSV (Control: 0.11 ± 0.02, RSV: 0.11 ± 0.01, VPA: 0.06 ± 0.01, RSV+VPA: 0.1 ± 0.007, *F* = 9.8; *p* = 0.0290 to VPA vs. RSV+VPA). When combining all layers, we only observed an effect of RSV (Figure 3Bi), reducing the PV^+^-neurons/total neurons ratio (Control: 0.10 ± 0.007, RSV: 0.08 ± 0.004, VPA: 0.11 ± 0.014, RSV+VPA: 0.09 ± 0.002, *F* = 7.5; *p* = 0.0438). Thus, our immunofluorescence data suggests a protective effect of RSV for the laminar organization and correct distribution of PV^+^-neurons in animals of the VPA model of autism.

### RSV Reestablishes a Typical Proportion of PV^+^-Neurons in the Amygdala

**Figure 4:**
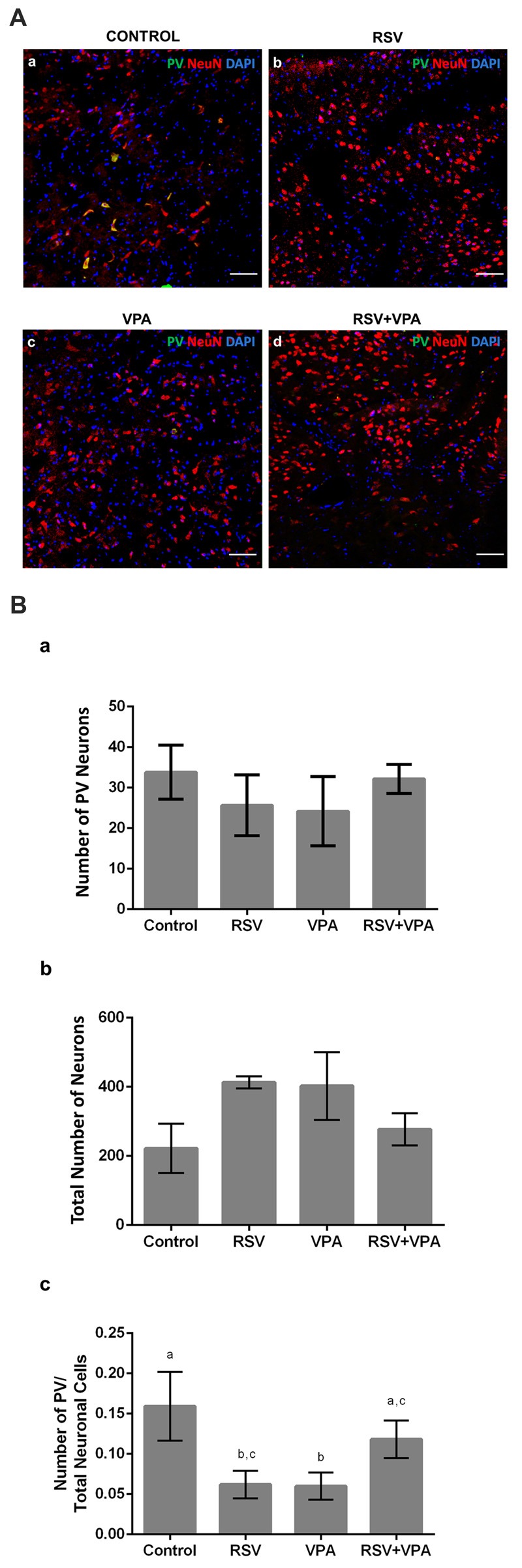
RSV restores the correct proportion of PV+ neurons in the amygdala of rats of the animal model of autism induced by VPA. Cell nuclei—blue (DAPI), total neurons—red (NeuN^+^) and GABAergic PV^+^ neurons—green (PV^+^). **(Aa–d)** Representative immunofluorescence images of the amygdala Region (AmR). Cell distribution and organization can be visualized along the basolateral portion of AmR.** (Ba–c)** Quantitative analysis of the density of PV^+^ neurons in the AmR. Different letters indicate statistically significant differences between groups. Mean ± SD were represented. Statistical analysis: ordinary one-way ANOVA followed by Bonferroni. Scale bar = 50 μm.

Given the importance of the AmR to attribute affective content to sensory information, we also evaluated the quantity of PV^+^-neurons in this region. Figures 4Aa–d shows illustrative micrographs from PV^+^-neuron distribution in the amygdala of our four experimental groups. As showed in Figure 4Ba, no significant differences were observed in the number of PV^+^-cells (Control: 33.8 ± 6.6, RSV: 25.6 ± 7.5, VPA: 24.2 ± 8.5, RSV+VPA: 32.1 ± 3.5, *p* = 26, *F* = 1.55) or (Figure 4Bb) in the total number of neuronal cells (Control: 221.8 ± 71.6, RSV: 413 ± 17.5, VPA: 402.6 ± 97.9, RSV+VPA: 277.3 ± 46.3, *p* = 0.2119, *F* = 1.79). Nevertheless, we observed a significant reduction in the PV^+^-neurons/total neurons ratio (Figure 4Bc) in the RSV (*p* = 0.0040) and VPA groups (*p* = 0.0015), when compared to the control group. Interestingly, RSV was able to totally prevent the VPA effect (Control: 0.16 ± 0.04, RSV: 0.06 ± 0.017, VPA: 0.059 ± 0.016, RSV+VPA: 0.12 ± 0.02, *F* = 12.09). Here, our results demonstrate interesting and complex effects of RSV and VPA exposures in the AmR in which the combined actions of VPA and RSV normalized the PV^+^/total neurons ratio.

### VPA and RSV Modulate Synaptic Proteins in Primary Somatosensory Area (PSSA) and Amygdala Region (AmR)

**Figure 5:**
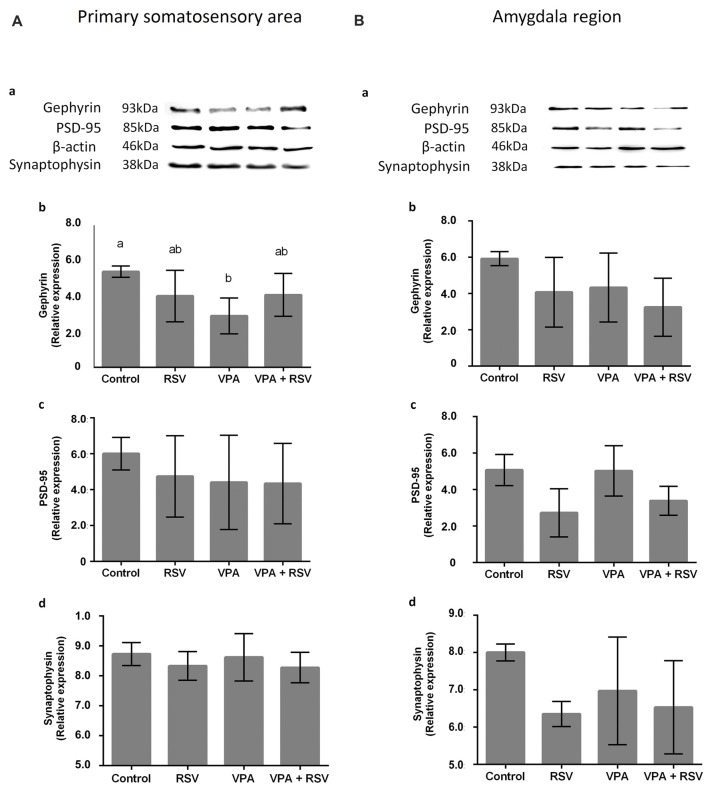
Effects of prenatal exposure to VPA and RSV in synaptic proteins in PSSA and AmR. Representative images of Western blotting for synaptic proteins are shown in **(Aa)** from PSSA and in **(Ba)** from AmR. Protein quantification of gephyrin, PSD-95 and synaptophysin are shown in **(Ab–d)** for PSSA and in **(Bb–d)** for AmR, respectively. Different letters indicate statistically significant differences between groups. Mean ± SD were plotted. Statistical analysis: ordinary One-Way ANOVA followed by Bonferroni.

Next, we asked if the neuronal reorganization promoted by VPA and prevented by RSV in PSSA and AmR influenced the overall expression of synaptic proteins. We evaluated proteins from excitatory (PSD-95) and inhibitory (gephyrin) synapses, as well as synaptophysin, an ubiquitous pre-synaptic component. Illustrative western blot images are shown in Figure 5Aa (PSSA) and Figure 5Ba for (AmR). No significant differences were observed between groups in PSD-95 levels in PSSA (Figure 5Ac) or AmR (Figure 5Bc). On the other hand, gephyrin levels were reduced in the PSSA of VPA animals compared to the control group (Control: 5.306 × 10^7^ ± 3.123 × 10^6^, RSV: 3.959 × 10^7^ ± 1.426 × 10^7^, VPA: 2.859 × 10^7^ ± 9.937 × 10^7^, RSV+VPA: 4.020 × 10^7^ ± 1.188 × 10^7^, *F* = 3.54; *p* = 0.0305). In the PSSA of RSV+VPA animals, gephyrin is expressed at intermediate levels between the control and VPA groups, not being statistically different from either one (Figure 5Ab). No significant differences were observed in the expression of gephyrin in the AmR of our experimental groups (Figure 5Bb). The synaptophysin and PSD-95 expression levels are not significantly different between groups in PSSA (Figure 5Ad) or AmR (Figure 5Bd).

## Discussion

The response to sensory stimuli is altered in more than 90% of ASD patients resulting in great impairment in synaptic transmission and processing, affecting the health of these individuals (Coskun et al., 2009; Geschwind, 2009). These alterations include hyper-responsiveness to auditory, visual and tactile stimuli and hypo responsiveness to nociceptive stimuli. An interesting hypothesis suggests a perturbation in the processing and integration of the sensory information between different areas, resulting in local and global alterations, from neurotransmitter release, to the neural network, leading to disruption of sensory perception (Coskun et al., 2013; Supekar et al., 2013). Here we report, for the first time, that RSV prevented the alterations caused by VPA in the WNT, a behavioral test that evaluates quality of response to direct whisker stimulation. In addition to the striking result in the present work, RSV was able to preserve the cortical laminar patterning and the distribution of PV^+^-neurons affected by VPA in the PSSA, the brain area related to tactile processing of the whiskers (Chen-Bee et al., 2012). These results corroborate similar findings in the literature in other brain regions involved in sensory processing, such as the superior colliculus, presenting dysfunctional sensory processing characterized by an inability to filter sensory information and impairments in GABAergic synaptic transmission, particularly simultaneously arriving multimodal inputs (Dendrinos et al., 2011).

The behavior evaluated in the NS test is of extreme importance for the development of rats, which need major care from their mothers to survive. This attachment behavior is present in many species (Broad et al., 2006) and combines a sensory component (olfactory, in rodents) with an affective memory, creating a mother-offspring connection that allows young animals (who can barely see or hear at this age) to find the source of food and protection. The AmR is an important area for primary attachment behaviors and other emotional responses, such as aversive behavior (Landers and Sullivan, 2012; Rigon et al., 2016). In the context of ASD, it is known that many alterations were already described in the amygdala, including hyper reactivity (Markram et al., 2008; Lin et al., 2013), enlargement and hyper cellularity (Markram et al., 2008; Ecker et al., 2015), elevated NMDA receptor levels and enhanced postsynaptic long-term potentiation (LTP; Rinaldi et al., 2007). Therefore, our hypothesis highlights the possibility that an alteration in this region is causing the impairment identified in the NS task and can be related to the outcomes in social development. Interestingly, studies have demonstrated that the AmR seems to develop faster in animals of the VPA model of autism. It results in premature maturation of fear responses, caused by the hyper reactivity, hyper plasticity and deficits in inhibitory system found in the lateral amygdala, which could lead to an early termination of the attachment learning period (Markram et al., 2008; Landers and Sullivan, 2012; Barrett et al., 2017).

We report in our present work that RSV reestablishes the typical proportion of GABAergic PV^+^-neurons in the amygdala of VPA animals, which might be crucial for proper inhibition of responses in the amygdala (McDonald and Betette, 2001; Woodruff and Sah, 2007; Bocchio et al., 2015). The majority of PV^+^-neurons originate in the medial ganglionic eminence and migrate to their target regions in the brain (Guo and Anton, 2014). The ganglionic eminence is a temporary brain structure first observed at E11.5 in rodents (Lavdas et al., 1999; Anderson et al., 2001; Marín et al., 2001) with the first interneurons starting their tangential migration towards the cortex at E12.5 in mice (Kelsom and Lu, 2013). This coincides with our hypothesis of the time point of VPA administration and opens the intriguing possibility that VPA interferes either with GABAergic interneuron specification and proliferation or their migration to the cortical layers and their survival in the cortex, promoting the increase of PV^+^-neuronal density in layer II/III and the decrease of this subpopulation in layer IV/V. In fact, studies with 7 days old mice of the VPA model of autism present a reduced number of BrdU^+^ cells (generated at E12.5) in the deep layers of the somatosensory and prefrontal cortices, indicating several impairments in cortical migration related to cortical areas (Kataoka et al., 2013).

A decreased number of PV^+^-neurons and of PV mRNA was observed in several animal models related to ASD (Gogolla et al., 2009; Wöhr et al., 2015; Lauber et al., 2016) and a recent study demonstrated reduction in the number of PV^+^-neuros in the medial prefrontal cortex of individuals with autism (Hashemi et al., 2017). Even though the significance of the reduction of PV^+^-neurons in ASD remains unclear, PV knockout mice display behavioral phenotypes related to all the core symptoms present in ASD patients, such as abnormal reciprocal social interactions, altered ultrasonic vocalization and presence of repetitive/stereotyped patterns of behavior (Wöhr et al., 2015). Moreover, there is an increase of apoptosis in the developing neocortex 12 h and 24 h after VPA exposure, accompanied by a reduction in proliferation in the ganglionic eminences (Kataoka et al., 2013). This can likely have widespread and long-lasting consequences to brain organization, since, during development, GABAergic neurons are excitatory and promote the maturation of neural networks (Le Magueresse and Monyer, 2013).

Finally, in western blotting analysis, we observed in this work a significant alteration in the expression level of the protein gephyrin (a key scaffolding protein of inhibitory synapses) in PSSA: VPA decreased its levels and RSV treatment was able to prevent this impairment. These findings corroborate previous studies showing an excitatory/inhibitory imbalance in cortical regions of animal models of ASD (Rinaldi et al., 2008; Gao and Penzes, 2015; Nelson and Valakh, 2015). Since, VPA affects the PV^+^-neuron localization and distribution, this might lead to altered inhibitory synaptic distribution and organization, as seen in autistic individuals (Zikopoulos and Barbas, 2013; Gao and Penzes, 2015; Nelson and Valakh, 2015). Thus, the RSV prevention could be related to the excitation/inhibition balance restoration in the PSSA, leading to correct sensory perception/processing.

It is also possible that both RSV and VPA primarily exert independent actions in the developing nervous system, modulating neuronal proliferation, migration and establishment of synaptic connections. It is worth to mention that RSV is itself, a teratogen. Therefore, we are not proposing this approach as a “vaccine” to ASD but as a potential research tool, that could help to clarify specific mechanisms related to ASD etiology and pathophysiology.

Our results from behavioral, histological and protein analyses showed a relevant impact of RSV treatment in sensory aspects of the VPA animal model of autism. Thus, RSV can be used as a tool to study pathways related to ASD pathophysiology, and further investigation of VPA effects counteracted by RSV can help to shed light in molecular mechanisms involved in the etiology of ASD.

## Concluding Remarks

Taken together, our data showed important deficits in the processing and integration of sensory information in the VPA animal model of autism, corroborating the face validity of this model. Furthermore, the prenatal treatment of RSV successfully prevented sensory deficits in behavioral analyses, possibly by correcting altered PV^+^-neuron localization and cortical organization impaired by VPA.

We suggest that not only the correct number, but also localization of PV^+^-neurons throughout the PSSA cortical layers, might play important roles in proper sensory processing, refining the excitatory inputs. Additionally, in AmR, the correct balance of this neuronal subpopulation might be necessary to attribute the correct emotional load to sensory information, providing a refined and complex cognitive experience. Thus, the perturbation of PV by VPA may be an important player in the sensory behavioral deficits evaluated.

Since RSV appears as a promising molecule for investigation of ASD etiology and pathophysiology, it is important to explore whether these effects result from its anti-inflammatory or anti-oxidant properties or from previously unrecognized activities of this compound. Furthermore, it will be of upmost importance to investigate the opposite actions of VPA and RSV during embryonic development and in the pregnant female to characterize the molecular alterations involved in the triggering of autistic-like alterations in the VPA animal model of autism. Again, we think that our data support the possible therapeutic use of RSV, but future studies have to be done to show if RSV has any beneficial effect on the postnatal development of animals presenting autistic-like features.

## Author Contributions

MF-D, CG, VB-J, RR and CH-P: experimental design and intellectual contribution. MF-D, JS-T, ID, GBS, GD-FN, MMH and GB-N: *in vivo* and *in vitro* analyses. MF-D, JS-T, ID, GBS, GD-FN, MMH, GB-N, CH-P, VB-J, RR and CG: data discussion and manuscript preparation.

## Conflict of Interest Statement

The authors declare that the research was conducted in the absence of any commercial or financial relationships that could be construed as a potential conflict of interest.
