# Supplementary material for: Resveratrol Prevents Cellular and Behavioral Sensory Alterations in the Animal Model of Autism Induced by Valproic Acid
Source: Front Synaptic Neurosci. 2018 May 22;10:9. doi: 10.3389/fnsyn.2018.00009 (PMC5972198; doi:10.3389/fnsyn.2018.00009)
Supplement: Supplementary file 1 [file Table_1.DOCX]

Supplementary Material

**Resveratrol prevents cellular and behavioral sensory alterations in the animal model of autism induced by valproic acid**

Mellanie Fontes-Dutra^1,2,3*^, Júlio Santos-Terra^1,2,3^, Iohanna Deckmann^1,2,3^, Gustavo Brum Schwingel^1,2,3^, Gustavo Della-Flora Nunes^1,3,4^, Mauro Mozael Hirsch^1,2,3^, Guilherme Bauer-Negrini^1,2,3^, Victorio Bambini-Júnior^1,3,6^, Rudimar Riesgo^1,3,7^, Cecília Hedin-Pereira^3,5,8^, Carmem Gottfried^1,2,3*^

1 Translational Research Group in Autism Spectrum Disorders-GETTEA, Universidade Federal do Rio Grande do Sul -UFRGS, 90035-003 Porto Alegre, RS, Brazil.

2 Department of Biochemistry, Universidade Federal do Rio Grande do Sul -UFRGS, 90035-003 Porto Alegre, RS, Brazil.

3 National Institute of Science and Technology on Neuroimmunomodulation -

INCT-NIM, Oswaldo Cruz Institute, Oswaldo Cruz Foundation, Rio de Janeiro,

Brazil.

4 Department of Biochemistry, University of Buffalo, The State University of New York, NY, USA

5 Institute of Biophysics Carlos Chagas Filho, Rio de Janeiro, RJ, Brazil

6 School of Pharmacology and Biomedical Sciences, University of Central Lancashire, PR1 2HE, Preston, UK

7 Child Neurology Unit, Clinical Hospital of Porto Alegre, Federal University of Rio

Grande do Sul, Porto Alegre, Brazil.

8 VPPCB – Oswaldo Cruz Foundation, Fiocruz, Rio de Janeiro, RJ, Brazil

*Corresponding authors:

Carmem Gottfried

[carmem.gottfried@gmail.com](mailto:carmem.gottfried@gmail.com)

Mellanie Fontes-Dutra

[dutra.mellanie@gmail.com](mailto:dutra.mellanie@gmail.com)

| Table 1S: Observed Behavioral Criteria Used in the Whisker Nuisance Task | *P1* | *P2* | *P3* |
| --- | --- | --- | --- |
| Freezing |  |  |  |
| Walking around, exhibits curious behavior | 0 | 0 | 0 |
| Slow *or* stationary, limited curiosity, cautious | 1 | 1 | 1 |
| Freezing, defensive, and fearful | 2 | 2 | 2 |
| Stance and body position |  |  |  |
| Relaxed, looking skyward, forepaws under body | 0 | 0 | 0 |
| Cowering, guarded, grounded forepaws | 2 | 2 | 2 |
| Breathing |  |  |  |
| Normal range | 0 | 0 | 0 |
| Forced, gasping | 2 | 2 | 2 |
| Whisker position |  |  |  |
| Fully protracted (both sides) | 0 | 0 | 0 |
| Protraction and retraction | 1 | 1 | 1 |
| Fully retracted (both sides) | 2 | 2 | 2 |
| Whisking response |  |  |  |
| Standard whisking, normal movement | 0 | 0 | 0 |
| Tremors, twitching | 1 | 1 | 1 |
| None, stopped | 2 | 2 | 2 |
| Evading stimulation |  |  |  |
| No evasive behavior | 0 | 0 | 0 |
| Escape behavior or directed movement to avoid/protect whiskers | 2 | 2 | 2 |
| Response to stick presentation |  |  |  |
| Ambivalence *or* curiosity about stick | 0 | 0 | 0 |
| Avoiding and anxiety *or* biting and attacking *or* freezing | 2 | 2 | 2 |
| Grooming |  |  |  |
| No, minimal, or normal grooming | 0 | 0 | 0 |
| Irritated scratching/rubbing/pulling | 2 | 2 | 2 |

The first column discriminates the observed behavioral analysis during the trials (Trial 1: P1; Trial 2: P2; Trial 3: P3, each one during 5 min). Normal behavioral responses to whisker stimulation are assigned a value of zero; abnormal responses were assigned a value of 2. A separate WNT score is obtained by the sum of each period and the final score is an average among the three trials for a single animal. The maximum total score an animal can receive is 16. Adapted from McNamara, *et al*., J Neurotrauma. 2010 Apr; 27(4): 695–706.
