# Supplementary material for: Resveratrol Prevents Cellular and Behavioral Sensory Alterations in the Animal Model of Autism Induced by Valproic Acid
Source: Front Synaptic Neurosci. 2018 May 22;10:9. doi: 10.3389/fnsyn.2018.00009 (PMC5972198; doi:10.3389/fnsyn.2018.00009)
Supplement: Supplementary file 2 [file Table_2.DOCX]

Supplementary Material

**Resveratrol prevents cellular and behavioral sensory alterations in the animal model of autism induced by valproic acid**

Mellanie Fontes-Dutra^1,2,3*^, Júlio Santos-Terra^1,2,3^, Iohanna Deckmann^1,2,3^, Gustavo Brum Schwingel^1,2,3^, Gustavo Della-Flora Nunes^1,3,4^, Mauro Mozael Hirsch^1,2,3^, Guilherme Bauer-Negrini^1,2,3^, Victorio Bambini-Júnior^1,3,6^, Rudimar Riesgo^1,3,7^, Cecília Hedin-Pereira^3,5,8^, Carmem Gottfried^1,2,3*^

1 Translational Research Group in Autism Spectrum Disorders-GETTEA, Universidade Federal do Rio Grande do Sul -UFRGS, 90035-003 Porto Alegre, RS, Brazil.

2 Department of Biochemistry, Universidade Federal do Rio Grande do Sul -UFRGS, 90035-003 Porto Alegre, RS, Brazil.

3 National Institute of Science and Technology on Neuroimmunomodulation -

INCT-NIM, Oswaldo Cruz Institute, Oswaldo Cruz Foundation, Rio de Janeiro,

Brazil.

4 Department of Biochemistry, University of Buffalo, The State University of New York, NY, USA

5 Institute of Biophysics Carlos Chagas Filho, Rio de Janeiro, RJ, Brazil

6 School of Pharmacology and Biomedical Sciences, University of Central Lancashire, PR1 2HE, Preston, UK

7 Child Neurology Unit, Clinical Hospital of Porto Alegre, Federal University of Rio

Grande do Sul, Porto Alegre, Brazil.

8 VPPCB – Oswaldo Cruz Foundation, Fiocruz, Rio de Janeiro, RJ, Brazil

*Corresponding authors:

Carmem Gottfried

[carmem.gottfried@gmail.com](mailto:carmem.gottfried@gmail.com)

Mellanie Fontes-Dutra

[dutra.mellanie@gmail.com](mailto:dutra.mellanie@gmail.com)

| Table 2S: Immunofluorescence reagents information | | | |  |
| --- | --- | --- | --- | --- |
| Reagent | Supplier | Code | Dilution/Volume | Batch |
| Anti-Parvalbumin | Abcam | Ab64555 | 1:500 | GR210362-1 |
| Anti-NeuN | Abcam | Ab177487 | 1:500 | GR173506-17 |
| Alexa Fluor 488 Goat Anti-Mouse IgG (H+L) | Molecular Probes | A11029 | 1:2000 | 1484573 |
| Alexa Fluor 546 Goat Anti-Rabbit IgG (H+L) | Molecular Probes | A11036 | 1:2000 | 1558746 |
| DAPI Nucleic Acid Stain | Invitrogen | MP01306 | 30 uL per slice | 113M4003V |
| Mounting Medium Fluorshield | Sigma-Aldrich | F6182-20ML | 30 uL per slice | #MKBT5415V |
