# Supplementary material for: Resveratrol Prevents Cellular and Behavioral Sensory Alterations in the Animal Model of Autism Induced by Valproic Acid
Source: Front Synaptic Neurosci. 2018 May 22;10:9. doi: 10.3389/fnsyn.2018.00009 (PMC5972198; doi:10.3389/fnsyn.2018.00009)
Supplement: Supplementary file 3 [file Table_3.DOCX]

Supplementary Material

**Resveratrol prevents cellular and behavioral sensory alterations in the animal model of autism induced by valproic acid**

Mellanie Fontes-Dutra^1,2,3*^, Júlio Santos-Terra^1,2,3^, Iohanna Deckmann^1,2,3^, Gustavo Brum Schwingel^1,2,3^, Gustavo Della-Flora Nunes^1,3,4^, Mauro Mozael Hirsch^1,2,3^, Guilherme Bauer-Negrini^1,2,3^, Victorio Bambini-Júnior^1,3,6^, Rudimar Riesgo^1,3,7^, Cecília Hedin-Pereira^3,5,8^, Carmem Gottfried^1,2,3*^

1 Translational Research Group in Autism Spectrum Disorders-GETTEA, Universidade Federal do Rio Grande do Sul -UFRGS, 90035-003 Porto Alegre, RS, Brazil.

2 Department of Biochemistry, Universidade Federal do Rio Grande do Sul -UFRGS, 90035-003 Porto Alegre, RS, Brazil.

3 National Institute of Science and Technology on Neuroimmunomodulation -

INCT-NIM, Oswaldo Cruz Institute, Oswaldo Cruz Foundation, Rio de Janeiro,

Brazil.

4 Department of Biochemistry, University of Buffalo, The State University of New York, NY, USA

5 Institute of Biophysics Carlos Chagas Filho, Rio de Janeiro, RJ, Brazil

6 School of Pharmacology and Biomedical Sciences, University of Central Lancashire, PR1 2HE, Preston, UK

7 Child Neurology Unit, Clinical Hospital of Porto Alegre, Federal University of Rio

Grande do Sul, Porto Alegre, Brazil.

8 VPPCB – Oswaldo Cruz Foundation, Fiocruz, Rio de Janeiro, RJ, Brazil

*Corresponding authors:

Carmem Gottfried

[carmem.gottfried@gmail.com](mailto:carmem.gottfried@gmail.com)

Mellanie Fontes-Dutra

[dutra.mellanie@gmail.com](mailto:dutra.mellanie@gmail.com)

| Table 3S: Western blotting antibodies information | | | | | | | | | | |  |
| --- | --- | --- | --- | --- | --- | --- | --- | --- | --- | --- | --- |
| Reagent | | Medium | | Dilution | | | Supplier | | | Time |  |
| Primary Antibodies | | | | | | | | | | |  |
| Anti-PSD95 | | | 5% BSA-TTBS 0,1% buffer | | 1:500 | | | abcam ab18258 | | Overnight at 4ºC | |
| Anti-Gephyrin | | | 5% BSA-TTBS 0,1% buffer | | 1:500 | | | abcam ab32206 | | Overnight at 4ºC | |
| Anti-Synaptophysin | | | 5% milk-TTBS 0,1% buffer | | 1:1000 | | | SIGMA Aldrich s5768 | | Overnight at 4ºC | |
| β-actin | | | TTBS 0,1% buffer | | 1:2000 | | | Cell Signaling (D6A8) | | Overnight at 4ºC | |
| Secondary Antibodies | | | | | | | | | | |  |
| Donkey anti-mouse IgG-HRP | 5% milk-TTBS 0,1% buffer | | | | | 1:1000 | | | Santa Cruz Biotechnology, INC., sc2314 | Room temperature for 1h | |
| Goat anti-rabbit IgG-HRP | TTBS 0,1% buffer | | | | | 1:2000 | | | Santa Cruz Biotechnology, INC., sc2004 | Room temperature for 1h | |
