# Supplementary material for: Resveratrol Prevents Cellular and Behavioral Sensory Alterations in the Animal Model of Autism Induced by Valproic Acid
Source: Front Synaptic Neurosci. 2018 May 22;10:9. doi: 10.3389/fnsyn.2018.00009 (PMC5972198; doi:10.3389/fnsyn.2018.00009)
Supplement: Supplementary file 4 [file Image_1.PDF]

Supplementary Material

Resveratrol prevents cellular and behavioral sensory alterations in the animal model of autism induced by valproic acid

Mellanie Fontes-Dutra[1,2,3*], Júlio Santos-Terra[1,2,3], Iohanna Deckmann[1,2,3], Gustavo Brum Schwingel[1,2,3], Gustavo Della-Flora Nunes[1,3,4], Mauro Mozael Hirsch[1,2,3], Guilherme Bauer-Negrini[1,2,3], Victorio Bambini-Júnior[1,3,6], Rudimar Riesgo[1,3,7], Cecília Hedin-Pereira[3,5,8], Carmem Gottfried[1,2,3*]

1 Translational Research Group in Autism Spectrum Disorders-GETTEA, Universidade Federal do Rio Grande do Sul -UFRGS, 90035-003 Porto Alegre, RS, Brazil.

2 Department of Biochemistry, Universidade Federal do Rio Grande do Sul -UFRGS, 90035-003 Porto Alegre, RS, Brazil.

3 National Institute of Science and Technology on Neuroimmunomodulation -

INCT-NIM, Oswaldo Cruz Institute, Oswaldo Cruz Foundation, Rio de Janeiro,

Brazil.

4 Department of Biochemistry, University of Buffalo, The State University of New York, NY, USA

5 Institute of Biophysics Carlos Chagas Filho, Rio de Janeiro, RJ, Brazil

6 School of Pharmacology and Biomedical Sciences, University of Central Lancashire, PR1 2HE, Preston, UK

7 Child Neurology Unit, Clinical Hospital of Porto Alegre, Federal University of Rio

Grande do Sul, Porto Alegre, Brazil.

8 VPPCB – Oswaldo Cruz Foundation, Fiocruz, Rio de Janeiro, RJ, Brazil

*Corresponding authors:

Carmem Gottfried

carmem.gottfried@gmail.com

Mellanie Fontes-Dutra

dutra.mellanie@gmail.com

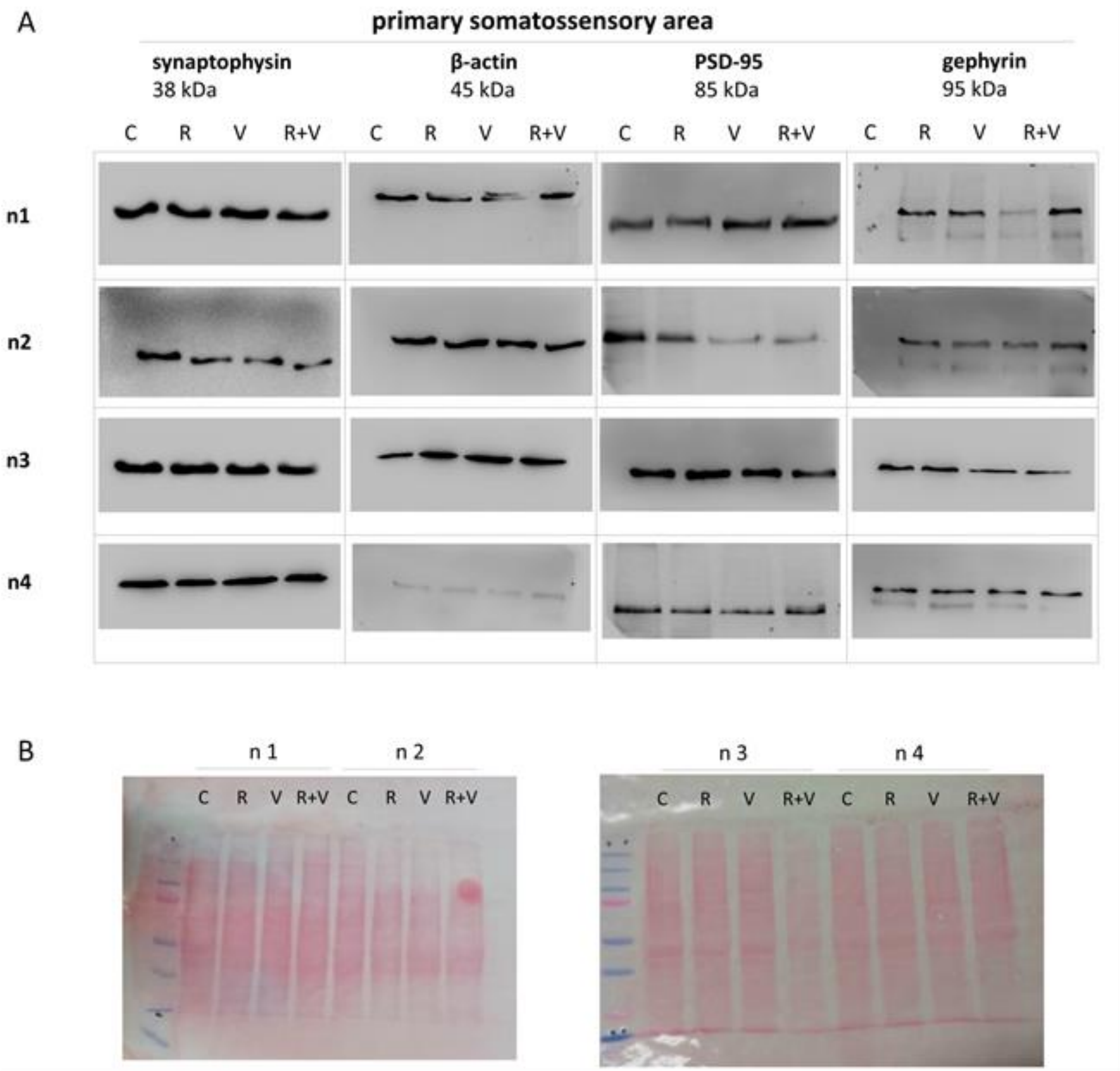

Figure 1S: Nitrocellulose membranes from primary somatosensory area (PSSA). A, n1, n2, n3, n4 represent membranes for animal 1, animal 2, animal 3 and animal 4, respectively. Columns represent protein-target (Synaptophysin, β-Actin, PSD-95 and Gephryn). The groups are discriminated as C for control group, R for RSV group, V for VPA group and R+V for RSV+VPA group. B, the nitrocellulose membranes stained with Ponceau.
